# Formal Anti‐Markovnikov Addition of Water to Olefins by Titanocene‐Catalyzed Epoxide Hydrosilylation: From Stoichiometric to Sustainable Catalytic Reactions

**DOI:** 10.1002/gch2.202200240

**Published:** 2023-05-02

**Authors:** Sebastian Höthker, Andreas Gansäuer

**Affiliations:** ^1^ Kekulé‐Institut für Organische Chemie und Biochemie Rheinische Friedrich‐Wilhelms‐Universität Bonn Gerhard‐Domagk‐Straße 1 53121 Bonn Germany

**Keywords:** catalysis, epoxides, green chemistry, hydrosilylation, sustainability, titanium

## Abstract

Here, the evolution of the titanocene‐catalyzed hydrosilylation of epoxides that yields the corresponding anti‐Markovnikov alcohols is summarized. The study focuses on aspects of sustainability, efficient catalyst activation, and stereoselectivity. The latest variant of the reaction employs polymethylhydrosiloxane (PMHS), a waste product of the Müller–Rochow process as terminal reductant, features an efficient catalyst activation with benzylMgBr and the use of the bench stable Cp_2_TiCl_2_ as precatalyst. The combination of olefin epoxidation and epoxide hydrosilylation provides a uniquely efficient approach to the formal anti‐Markovnikov addition of H_2_O to olefins.

## Graphical abstract

This ten‐year retrospective shows the development of the titanocene‐catalyzed epoxide hydrosilylation as an alternative, more sustainable approach to the hydroboration/oxidation sequence toward anti‐Markovnikov alcohols. It distinguishes itself by perfect regio‐ and excellent stereoselectivity, as well as by the exclusive use of non‐toxic reagents based on earth‐abundant elements that even find application as food additives.

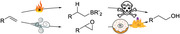

## 1 Introduction

The art of modern chemistry is steadily transforming from a purely target‐molecule‐oriented field to an area of research requiring continuous improvements to the process of synthesis with special regard to its environmental impact. To assist chemists in achieving this goal, the scientific community led by P. Anastas and M. Kirchhoff developed the “12 Principles of Green Chemistry”, which mostly target the safety and potential pollution of chemical processes.^[^ ^1^ ^]^ Especially in the latter regard, they elaborate on atom^[^ ^2^ ^]^ and energy efficiency,^[^ ^3^ ^]^ as well as the need for biodegradability or biological inertness of reactants and products.^[^ ^4^ ^]^ The major field of development in the realms of atom and energy efficiency is catalysis.^[^ ^5^ ^]^ Catalysts are to be employed in substoichiometric amounts and result in an acceleration of reactions because they lower activation energies and thus accelerate reactions. Generally, this reduces the potential material and energy consumption compared to a non‐catalyzed process.

It only takes a short look at the list of Nobel Prizes awarded for chemistry to realize that a world without our knowledge of catalysis would be vastly different. A selection of examples include the Haber–Bosch process forming ammonia,^[^ ^6^ ^]^ which is a major contributor to successfully feeding eight billion people, the Ziegler‐polymerisation^[^ ^7^ ^]^ yielding polyethylene and polypropylene and Pd‐catalyzed cross‐couplings,^[^ ^8^ ^]^ which are of immeasurable importance for the pharmaceutical^[^ ^9^ ^]^ and agrochemical industry.^[^ ^10^ ^]^

**Figure 1:**
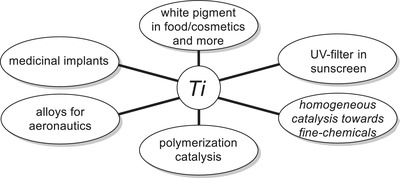
Selected examples of the use of titanium and its complexes.^[^ ^12^, ^13^, ^14^, ^15^, ^16^ ^]^

While catalysis is undeniably a key method for addressing the issues highlighted by the principles of Green Chemistry, a significant number of catalytic processes rely on the use of precious transition or rare earth metals, which are exceptionally scarce on our planet.^[^ ^11^ ^]^ It is therefore of large interest to develop catalytic reactions relying on earth‐abundant elements. Amongst the earth‐abundant transition metals, Titanium is one of the most interesting candidates for the development of catalytic methods as it is the second most abundant transition metal only to iron and the ninth most abundant element in the earth's continental upper crust.^[^ ^11^ ^]^ Its terminal oxidation product is titanium dioxide, which is inherently non‐toxic and thus in line with the desired biologically inert properties of a catalyst.^[^ ^12^ ^]^ While it is most known as a pigment in white paint, it is even commonly used as a whitening agent in food and cosmetics up to 1 mass% underlining its low‐risk potential.^[^ ^13^ ^]^ Furthermore, Titanium is valued as a component in alloys for medicinal,^[^ ^14^ ^]^ aeronautic, and military purposes^[^ ^15^ ^]^ as well as a powerful polymerization catalyst (**Figure** **1**).^[^ ^16^ ^]^

**Scheme 1:**
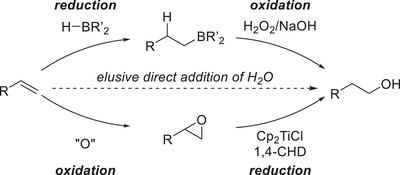
Comparison of hydroboration/oxidation versus epoxidation/reduction sequence.^[^ ^17^, ^26^ ^]^

Having these aspects in mind, our group set out to develop titanium(III)‐catalyzed reactions as substitutes to methodologies previously relying on stoichiometric quantities of rare elements. A prime example of this kind of transformation is the classical synthesis of anti‐Markovnikov alcohols via a hydroboration‐oxidation sequence employing overstoichiometric amounts of boranes.^[^ ^17^ ^]^ While boron‐containing compounds are common additives in detergents^[^ ^18^ ^]^ suggesting their ubiquity, boron is in fact comparably rare, as it only accounts for 11 ppm of the earth's upper continental mantle.^[^ ^11^ ^]^ Additionally, its recovery from water is also far from trivial.^[^ ^19^ ^]^ An alternative, boron‐free approach toward anti‐Markovnikov alcohols was developed in the 1990s by Nugent and RajanBabu based on a titanium(III)‐mediated reduction of epoxides.^[^ ^20^ ^]^ While both the hydroboration/oxidation as well as the epoxidation/titanocene‐mediated epoxide opening sequence are two‐step approaches from alkenes, they differ in their order of principal chemical transformations. In the hydroboration/oxidation sequence the alkene is initially reduced by a borane, before oxidizing the intermediate boranes by H_2_O_2_. Compared to that, the titanium‐based approach oxidizes the alkene first by epoxidation and subsequently reduces the formed epoxide to form the less substituted alcohol. While a direct catalytic addition of H_2_O in an anti‐Markovnikov fashion would be the most atom‐economical process, it remains an elusive transformation to this day. Rare examples often include high catalyst loadings of expensive and toxic late transition metals, such as Ruthenium and Palladium.^[^ ^21^ ^]^ Arguably, the most important industrial process toward anti‐Markovnikov alcohols is the hydroformylation‐reduction of simple olefins obtained from steam cracking. However, alongside the pitfall of elongating the carbon chain by one atom, the reaction can often also suffer from regioselectivity problems, which are less relevant on an industrial scale as usually both isomers are of value.^[^ ^22^ ^]^ For late‐stage functionalization, this is usually not the case and thus, the regioselectivity issues paired with high temperatures and pressures often prevent its application in these cases (**Scheme** **1**).

Epoxides are highly versatile building blocks that can be prepared in numerous different ways^[^ ^23^ ^]^ including industrial‐scale synthesis via heterogeneous catalysis.^[^ ^24^ ^]^ Their reactivity usually originates from their high ring strain (≈27 kcal mol^−1^)^[^ ^25^ ^]^ allowing the generation of highly reactive species, such as C‐centered radicals, which may undergo follow‐up reactions exemplified by (intramolecular) addition^[^ ^20^, ^26^ ^]^ or saturation via hydrogen atom transfer (HAT).^[^ ^20^, ^27^ ^]^ Despite their reactivity being commonly perceived as very high, epoxides often require electrophilic activation prior to ring opening, rendering them tolerant to a diverse range of conditions.^[^ ^28^ ^]^

**Scheme 2:**
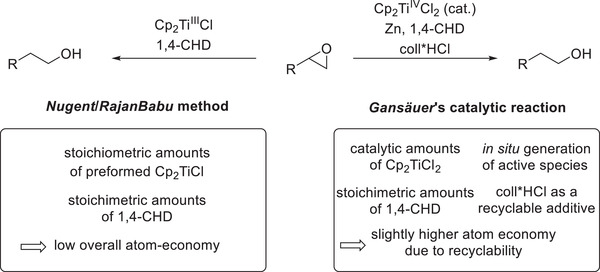
Comparison of the stoichiometric Nugent/RajanBabu system with Gansäuer's catalytic reaction.^[^ ^20^, ^26^ ^]^

By employing Cp_2_TiCl as a stoichiometric reagent, which can easily be prepared via reduction of the bench stable precatalyst Cp_2_TiCl_2_ using base metals such as zinc or manganese, and 1,4‐cyclohexadiene (1,4‐CHD) as a HAT (hydrogen atom transfer) reagent, epoxides are successfully converted to their respective less substituted alcohols in perfect regioselectivity.^[^ ^20c,d^ ^]^ Gansäuer and co‐workers were subsequently able to render the reaction catalytic in titanium by exploiting the catalyst's stability toward mild Brønsted acids, which alleviate the issue of product inhibition, thus leading to a catalytic turnover.^[^ ^29^ ^]^ In the past 20 years or so, numerous variants of catalytic epoxide openings have been developed including photo‐^[^ ^30^ ^]^ and electrochemical methods,^[^ ^31^ ^]^ as well as epoxide hydrogenation reactions via cooperative catalysis.^[^ ^32^ ^]^ Furthermore, radicals obtained from epoxides via single electron addition from titanium(III), have been employed in radical additions (including cyclizations)^[^ ^33^ ^]^ as well as in cross coupling reactions via cooperative catalysis (**Scheme** **2**).^[^ ^34^ ^]^

On the occasion of its tenth anniversary, we want to draw attention to the titanium‐catalyzed hydrosilylation as the pivotal regiodetermining step in the synthesis of anti‐Markovnikov alcohols.^[^ ^35^ ^]^ While the Ti/Si pair has been successfully employed in the reduction of amides,^[^ ^36^ ^]^ ketones,^[^ ^37^ ^]^ esters,^[^ ^38^ ^]^ imines,^[^ ^39^ ^]^ and enamines^[^ ^40^ ^]^ including some enantioselective methods in the 1990s, titanium‐catalyzed epoxide hydrosilylations have only been realized as recently as 2012.

**Figure 2:**
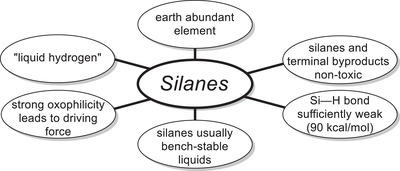
Advantageous qualities of silanes as HAT reagents. ^[^ ^41^, ^42^, ^46^, ^47^, ^48^ ^]^

Silanes are attractive terminal HAT reagents for various reasons. On the one hand, the Si–H bond is sufficiently weak (≈90 kcal mol^−1^)^[^ ^41^ ^]^ to act as a hydrogen donor while on the other hand, it is strong enough to be handled safely. Most silanes are bench‐stable liquids and due to their property to form elemental hydrogen upon exposition to certain metal catalysts, they have been dubbed “liquid organic hydrogen carriers (LOHCs)” leading to them occasionally being referred to as “liquid hydrogen”.^[^ ^42^ ^]^ The fact that silicon is additionally the second most abundant element on our planet^[^ ^11^ ^]^ renders the use of silanes advantageous over any other HAT reagent without even discussing concerns regarding their toxicities^[^ ^43^ ^]^ or the ones associated with reagents employed in parallel.^[^ ^44^ ^]^ Moreover, silanes are valued as sustainable polymer coatings for their biodegradability, hydrophobicity, and non‐toxicity.^[^ ^45^, ^46^ ^]^ Albeit the most environmentally friendly and atom‐economical HAT reagent would be hydrogen gas, which has also been successfully employed in radical epoxide openings via cooperative catalysis,^[^ ^32^ ^]^ hydrosilylation reactions are usually characterized by having a larger driving force^[^ ^47^ ^]^ due to the formation of strong Si—O bonds^[^ ^48^ ^]^ and cleavage of the weaker Si—H bonds.^[^ ^41^ ^]^ Additionally, silanes are significantly easier to handle, as working with pressurized hydrogen gas requires specialized equipment. The combination of aforementioned properties makes silanes highly attractive HAT reagents with respect to Green Chemistry (**Figure** **2**).

## 2 First Development on Titanocene‐Catalyzed Hydrosilylation of Epoxides

The first work on titanocene‐catalyzed epoxide hydrosilylations was published in 2012 and proposed two similar catalytic methodologies differing in the employed precatalysts.^[^ ^35^ ^]^ While the first variant relied on the light‐, air‐, and water‐sensitive dimeric Ti^III^ complex [Cp_2_Ti(OEt)]_2_ for active catalyst generation, the second version made use of Cp_2_TiMe_2_, which is more tolerant to air and water yet still light sensitive and thus has to be freshly prepared on a regular and frequent basis.

**Figure 3:**
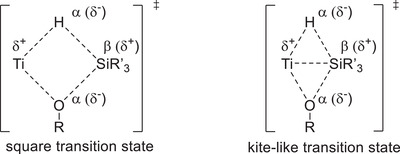
Proposed transition state structures for *σ*‐CAM.^[^ ^50^ ^]^

Regardless of the employed precatalyst, in situ generation of the catalytically active species using silanes is required. The active catalyst in titanocene‐catalyzed hydrosilylation reactions is in fact a titanocene(III) hydride (Cp_2_TiH) as opposed to other Ti^III^ mediated or catalyzed epoxides openings that rely on halides or sulfonates as anionic ligands.^[^ ^49^ ^]^ Precatalyst activation of [Cp_2_Ti(OEt)]_2_ constitutes a complex assisted *σ*‐bond metathesis (*σ*‐CAM)^[^ ^50^ ^]^ between the monomeric Cp_2_Ti(OEt) and Ph(Me)SiH_2_ exchanging the weak Si–H for the strong Si–O bond. *σ*‐CAM reactions are a prominent way for d^0^ or cationic metal complexes to perform ligand exchange since they are unwilling to perform oxidative addition/reductive elimination sequences. This concerted transformation is often regarded as a [2_*σ*_+2_*σ*_] cycloaddition. While this would be a formally symmetry‐forbidden process,^[^ ^51^ ^]^ the availability of vacant orbitals on the metal center eases the symmetry requirement. *σ*‐CAM processes are preferably occurring if the four‐membered transition state shows opposing polarization on neighboring corners, which is given for the *σ*‐CAM of a titanocene alkoxide with a silane. While the transition state had first been assumed to be square planar,^[^ ^50c^ ^]^ calculations have suggested a more kite‐like structure due to the initial “side‐on” coordination of the silane (**Figure** **3**).^[^ ^50d^ ^]^

Precatalyst activation of Cp_2_TiMe_2_ may require heating or even UV irradiation to induce homolytic bond fission in order to generate the desired Ti^III^ species. The exact mechanism of this activation has not been resolved yet.

Following the generation of Cp_2_Ti–H, it is able to bind the epoxide substrate forming a complex analogous to the cyclopropylmethyl radical, whose homolytic ring fragmentation is regularly being used to measure kinetics in radical reactions (radical clocks).^[^ ^52^ ^]^ Similarly, the titanocene(III) opens the epoxide^[^ ^20^ ^]^ to form the higher‐substituted and thus more stabilized radical^[^ ^53^ ^]^ oxidizing the titanium center from oxidation state +III to +IV. Therefore, this step can be viewed as a single‐electron oxidative addition.^[^ ^54^ ^]^ While two‐electron oxidative addition is a well‐established reaction in noble‐metal catalyzed C–X insertions, Ti^III^ complexes are unable to do the same as they contain only d^1^‐metal centers.

Following single‐electron oxidative addition, the resulting C‐centered radical is saturated by an intramolecular HAT from the titanocene(IV) hydride. As in this step, only a hydrogen atom is transferred, a single electron remains at the titanium center, yielding a [Ti(III)] complex. This renders the use of additional external reducing agents such as base metal powders, which are essential in most titanocene‐catalyzed epoxide reductions, superfluous. Additionally, the intramolecular nature of the HAT opens up the possibility for catalyst‐controlled stereoselectivity of radical reduction. If epoxides with a cyclic backbone are used as substrates, the HAT occurs almost exclusively from the same side the former epoxide was bound to. DFT (density functional theory) calculations suggest a five‐membered cyclic transition state with the activation barrier for *syn*‐selective HAT being approximately 3 kcal mol^−1^ lower than for the anti‐selective one. If intermolecular HAT reagents such as 1,4‐cyclohexadiene are used instead the selectivity is significantly inferior yielding the two diastereomeric products in a 65:35 mixture favoring the alcohol of *anti*‐selective HAT. Albeit the titanocene‐catalyzed hydrosilylation yields the desired anti‐Markovnikov alcohols in perfect regio‐ and excellent diastereoselectivity when applied to epoxides on cyclic scaffolds, it displays an even more remarkable reactivity when applied to epoxides that allow for rotation of the former epoxide C—C bond following single‐electron oxidative addition. This rotation allows for the interconversion of two diastereomeric radicals prior to HAT making it a prime example of Curtin–Hammet kinetics.^[^ ^55^ ^]^ As the *syn*‐selective HAT is slower than *σ*‐bond rotation the reaction is no longer only diastereoselective but diastereoconvergent.

By enlarging the catalyst's steric bulk the selectivity could be drastically improved from 85:15 to 97:3. The methodology's diastereoconvergence was showcased by comparing the performance of an equimolar mixture of diastereomeric epoxides and of a highly diastereomerically enriched epoxide under the catalytic conditions. The diastereoselectivites obtained were identically high (97:3) with similar yields proving the interconversion of *β*‐titanoxy radicals.

**Scheme 3:**
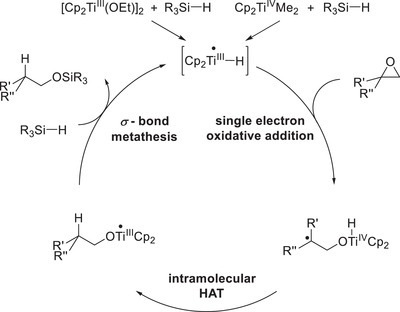
Catalytic cycle of the first‐generation epoxide hydrosilylation.^[^ ^35^ ^]^

**Scheme 4:**
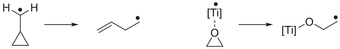
Isolobality of the cyclopropylmethyl radical and the [Ti^III^]‐epoxide complex.^[^ ^20^ ^]^

Succeeding radical saturation via intramolecular HAT, active catalyst liberation is essential to ensure catalytic turnover. Gratifyingly, the employed silane acts as a liberating agent as it may undergo *σ*‐CAM (comparable to initial active catalyst formation) with the Ti^III^‐alkoxide forming the desired titanocene(III)‐hydride and the alcohol trapped as a siloxane (**Scheme** **3**, **4** ). Desilylation under alkaline, aqueous conditions after completion of the reaction then cleaves the Si—O bond yielding the free alcohol (Scheme 4).

This mechanism shows that the silane is much more than merely a simple terminal HAT reagent. Instead, it is additionally responsible for catalyst reduction and is key to prevent product inhibition (**Scheme** 3, 5 ).

**Scheme 5:**
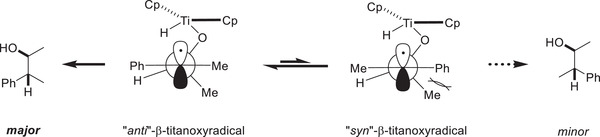
Rotation around C–C allowing for interconversion of diastereomeric radicals.^[^ ^35^ ^]^

**Scheme 6:**
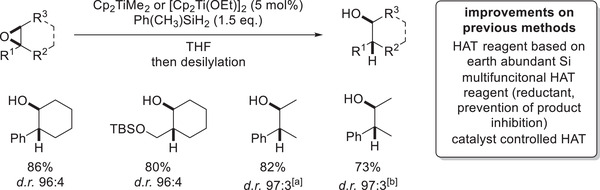
Selected examples of the first‐generation epoxide hydrosilylation; ^[a]^substrate *d.r*. 95:5; ^[b]^substrate *d.r*. 50:50.^[^ ^35^ ^]^

Using this methodology, a series of differently substituted epoxides can be converted to the respective less‐substituted alcohols including 1,1‐ and 1,2‐disubstituted as well as trisubstituted epoxides. Remarkably, the reaction conditions and in particular desilylation, could be applied to a TBS (tertbutyldimethyl silyl)‐protected primary epoxyalcohol. The secondary alcohol resulting from the epoxide moiety was cleanly liberated without deprotection of the primary hydroxy‐function (**Scheme** **6**).^[^ ^35^ ^]^

## 3 “Allyl Activation” Assisting in Facile Active Catalyst Generation

**Scheme 7:**
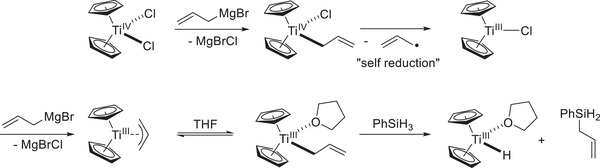
Allyl activation toward in situ generation of Cp_2_Ti–H.^[^ ^56^, ^57^, ^58^ ^]^

The goal for the development of the second‐generation titanocene‐catalyzed epoxide hydrosilylation was to avoid the use of sensitive precatalysts, whose activation proved to be capricious at times. The key steps of catalyst activation are the reduction of Ti^IV^ to Ti^III^ and the delivery of a hydride ligand to the metal center. Gratifyingly, Martin and Jellinek had reported the generation of an allyl‐titanocene(III) complex from commercially available titanocene(IV)dichloride and two equivalents of allylMgBr.^[^ ^56^ ^]^ Salt metathesis reactions lead to a ligand exchange of chloride for allyl ligands. Subsequent homolytic Ti—C bond fission yields a stabilized allyl radical and a titanocene(III)‐allyl complex. While this metallocene is *η* ^3^‐coordinated in the solid state, donor ligands and solvents have been postulated to induce *η* ^1^‐coordination.^[^ ^57^ ^]^ DFT calculations on *σ*‐CAM reactions of different titanocene(III) organyls and silanes, have indicated the reaction to be essentially thermoneutral for allyl‐titanocenes, whereas methyl‐titanocenes led to exergonic and phenyl‐titanocenes led to endergonic reactions. This places the stability of allyl‐titanocenes in an optimal position as they are stable enough to be handled safely and reliably but they are simultaneously reactive enough to perform efficient *σ*‐CAM to deliver the active titanocene(III) hydride (**Scheme** **7**).^[^ ^58^ ^]^

**Scheme 8:**
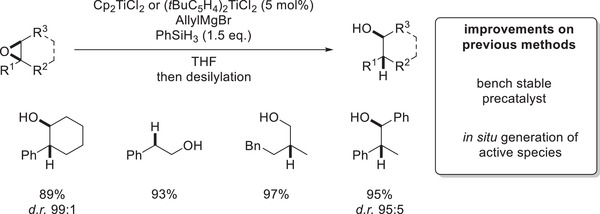
Selected examples of epoxide hydrosilylation via allyl activation.^[^ ^58^ ^]^

The activation process can be followed visually in a very convenient manner as the addition of allylMgBr turns the formerly red Cp_2_TiCl_2_ mixture to a dark‐violet solution of Cp_2_Ti^III^Allyl, while the subsequent addition of phenylsilane turns it into a green solution of Cp_2_Ti^III^H. Using this “allyl activation” for active catalyst generation, essentially identical to even better results to the first generation epoxide‐hydrosilylation could be achieved by employing bench‐stable precatalysts (**Scheme** **8**).

**Scheme 9:**
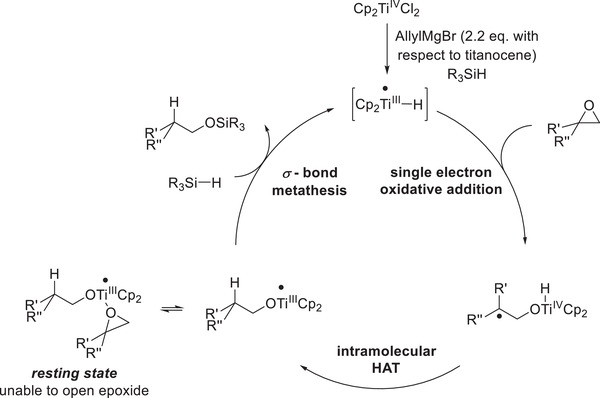
Catalytic cycle of epoxide hydrosilylation via allyl activation.^[^ ^58^ ^]^

Further mechanistic insights were obtained by elucidation of the reaction kinetics, which were revealed to be first order in titanocene, zeroth order in silane, and inverse first order in epoxide. This was rationalized by an off‐cycle resting state featuring epoxide coordination to a titanocene(III)‐alkoxide (**Scheme** **9**). Computational studies supported this postulated resting state as the reaction barrier for single‐electron oxidative addition is around 9 kcal/mol higher for titanocene alkoxides compared to the respective hydrides. The calculation is in agreement with the observation that [Cp_2_Ti(OEt)]_2_ does not open epoxides by itself.

## 4 Precision Deuteration via Epoxide Deuterosilylation: More Than a Simple Mechanistic Study

**Scheme 10:**
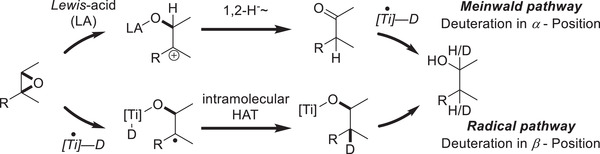
Conceivable pathways toward homobenzylic alcohols from epoxides.^[^ ^59^, ^60^ ^]^

The development of the third generation of titanocene‐catalyzed hydrosilylation reactions started out as an anticipated simple extension of the previous studies. Deuterated silanes were supposed to be used in order to unequivoqually prove the radical mechanism of the overall reaction as there has been a significant amount of work on *Lewis*‐acid‐catalyzed *Meinwald*‐rearrangements to give aldehydes or ketones, which are subsequently reduced by hydride or hydrogen atom donors.^[^ ^59^ ^]^ While this sequence usually leads to diastereomeric ratios that are significantly lower than observed for the titanocene‐catalyzed hydrosilylation, a deuteration study would indisputably prove the radical mechanism, as it would only give deuterium incorporation *β* to the alcohol whereas the *Meinwald*‐rearrangement/reduction sequence would exclusively yield *α*‐deuterated alcohols (**Scheme** **10**).

**Scheme 11:**
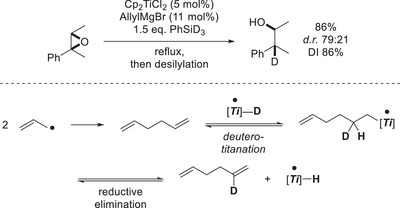
First results of epoxide deuterosilylation and reasoning behind low deuterium incorporation.^[^ ^60^ ^]^

However, deuterosilylation is not as straightforward as was initially assumed. A simple substitution of the silane for a deuterated silane gave very low yields under the first‐generation hydrosilylation conditions and while the allyl activation method yielded the products in high yields and high selectivity of deuterium incorporation (DI) in the *β*‐position, the DI was significantly lower than expected as it amounted to only around 90% (DI silane 98%). This behavior is attributable to the side product of the allyl‐activation 1,5‐hexadiene. This diene is able to undergo deuterotitanation/reductive elimination sequences leading to isotope scrambling on the titanium center reducing the overall DI in the organic product (**Scheme** **11**).^[^ ^60^ ^]^

**Scheme 12:**
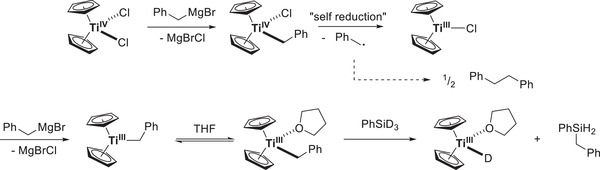
Benzyl activation toward isotope‐scrambling‐free in situ generation of Cp_2_Ti–D.^[^ ^60^ ^]^

This problem was circumvented by the development of the “benzyl‐activation”, which is closely related to the allyl‐activation but uses benzylMgBr instead. The byproduct bibenzyl is lacking olefinic double bonds and is thus unable to participate in isotope scrambling (**Scheme** **12**). Indeed deuterosilylation under the benzyl‐activation conditions usually leads to DIs of 98% with perfect regioselectivity of deuteration in the *β*‐position, excluding the *Meinwald*‐rearrangement/reduction pathway.

**Scheme 13:**
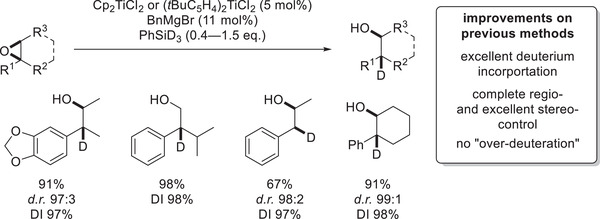
Selected examples of epoxide deuterosilylation.^[^ ^60^ ^]^

The titanocene‐catalyzed deuterosilylation's excellent regio‐ and stereoselectivity as well as its remarkable DI should empower it to be significantly more than a simple mechanistic proof as it should also be well suited to develop deuterated analogs of selected pharmaceuticals. Deuterated isotopomers have been found to show improved pharmacokinetic properties as their metabolism by P450 enzymes is often slowed down relative to their non‐deuterated counterpart.^[^ ^61^ ^]^ This behavior can be attributed to the increased stability of the C—D over the C—H bond, leading to an increased reaction barrier for homolytic bond fission, which is a key step in the P450 enzymes’ mode of action. In this regard, the deuteration of traditionally weak C–H bonds is of elevated interest as these positions are “soft‐spots” for metabolic oxidation. While there has been extensive research on C–H‐exchange reactions often relying on “perdeuteration”‐approaches that use an excess of deuterium source (usually as a solvent or D_2_), these reactions often lead to non‐quantitative deuterium incorporation in a multitude of positions.^[^ ^62^ ^]^ In contrast to that, the “precision deuteration” via titanocene‐catalyzed deuterosilylation leads to deuterium incorporation in a single position, in a well‐stereodefined way, and with almost quantitative DI. It is also exceptionally well suited in transferring D to benzylic positions,^[^ ^60^ ^]^ which are well‐known for having comparatively weak C—H bonds^[^ ^53b^ ^]^ making them one of the aforementioned soft‐spots for metabolic oxidation (**Scheme** **13**).^[^ ^61e^ ^]^

## 5 PMHS: A Terminal HAT Reagent In Line with the Principles of Green Chemistry

The latest publication on titanocene‐catalyzed hydrosilylations as of publishing this review deals with the use of polymethylhydrosiloxane (PMHS) as a terminal HAT reagent.^[^ ^63^ ^]^ PMHS is a very attractive silane as it arises as a by‐product of the Müller–Rochow process that is a widespread industrial process providing approximately 90% of raw materials for the manufacturing of silicones.^[^ ^64^ ^]^ As PMHS is a “waste‐product” its use as a reagent is highly attractive in the eyes of Green Chemistry because part of the overall waste from the Müller–Rochow process and the reaction employing PMHS can be shared between them reducing the ecological footprint of both processes. Since it is only a by‐product of a common industrial process it also has the advantage that it is up to two orders of magnitude less expensive than comparable HAT reagents.^[^ ^63^ ^]^

**Scheme 14:**
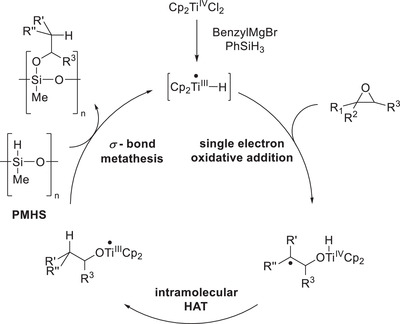
Catalytic cycle of PMHS‐mediated epoxide hydrosilylation.^[^ ^63^ ^]^

PMHS can indeed be used as a terminal HAT reagent for epoxide hydrosilylations under slightly altered conditions compared to the allyl‐ or benzyl‐activation methodologies. Firstly, PMHS is not reactive enough to perform the initial *σ*‐CAM with the benzyl‐titanocene(III). Thus, a small amount of “kickstarter‐PhSiH_3_” has to be used (0.5 eq. with respect to the catalyst) forming the initial [Cp_2_Ti–H]. Following single‐electron oxidative addition and intramolecular HAT as in previous methodologies, the obtained titanocene(III)‐alkoxide is then able to perform *σ*‐CAM with PMHS (**Scheme** **14**). In this case, the Si–O bond provides a larger driving force for *σ*‐CAM compared to the initial catalyst formation, in which a weaker Si–C bond is formed. Secondly, the choice of solvent is essential for achieving satisfying conversion. While THF (tetrahydrofuran), which is the standard solvent for previous iterations of epoxide hydrosilylations, as well as 1,4‐dioxane both lead to conversions of less than 20%, a 1:1 mixture of these two solvents allows for the isolation of the desired product in 82% yield. The addition of 1,4‐dioxane to epoxide hydrosilylation reactions in THF had previously been shown to increase their selectivity when applied to 1,1‐disubstituted epoxides due to the precipitation of Mg‐salts originating from the benzyl‐activation.

The PMHS‐based variant is, however, more than a “greener” variant of previous methodologies. While employing PhSiH_3_ or Ph(Me)SiH_2_ as terminal HAT reagents required the use of the more elaborate precatalyst (*t*BuC_5_H_4_)_2_TiCl_2_, high diastereomeric ratios of the desired alcohols can be obtained with simple Cp_2_TiCl_2_ tolerating a range of *ortho*‐, *meta*‐ and *para*‐substituted aromatics. In theory, even PMHS‐mediated deuterosilylations could be attainable via this method, due to effective H/D‐exchange reactions of silanes.^[^ ^65^ ^]^

**Scheme 15:**
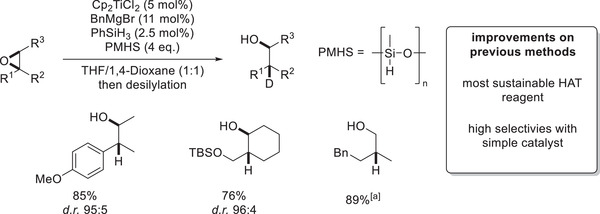
Selected examples of epoxide hydrosilylation employing PMHS as a terminal HAT reagent; ^[a]^5.0 g scale, catalyst loading reduced to 2.5 mol%.^[^ ^63^ ^]^

Considering the low cost of PMHS, the reaction is very feasible for upscaling that was demonstrated by an epoxide hydrosilylation on a 5.0 g scale in 89% yield while reducing the catalyst loading from 5% to 2.5% (**Scheme** **15**).

In terms of industrial applications, the synthesis of phthalate plasticizers such as Bis(2‐ethylhexyl) phthalate (DEHP), which are typically obtained by nucleophilic attack of alcohols on phtalic anhydride, may serve as one of the most interesting targets. In the case of DEHP, the required alcohol 2‐ethylhexanol is industrially produced via a sequence of hydroformylation, aldol condensation and reduction reactions starting from propene as a petroleum based feedstock.^[^ ^66^ ^]^ Due to DEHPs application as an additive for PVC, it is annually produced on a megaton scale.^[^ ^67^ ^]^ Thus, finding a renewable source of raw materials is certainly of elevated interest. In this regard, biobased butanol has been employed in a two‐step sequence of dehydration and oligomerization to provide 2‐ethyl‐1‐hexene on a >150 g scale.^[^ ^68^ ^]^ Subsequently, epoxidation and titanocene‐catalyzed epoxide opening using PMHS as a terminal HAT reagent, might provide the desired plasticizer alcohol in a highly sustainable process.

## 6 Summary

We have shown how a sustainable, catalytic, and stereoselective method for the opening of epoxides to anti‐Markovnikov alcohols could be developed from a stoichiometric reaction. The catalytic reaction is highly attractive with respect to sustainable chemistry because it employs a titanium‐based catalyst and not a noble metal catalyst, silanes, including PMHS, as terminal reductants, and epoxides as readily available starting materials. Moreover, together with olefin epoxidation, our method provides a simple and efficient method for the *anti*‐Markovnikov addition of water to olefins that is superior to the hydroboration‐oxidation sequences.

We note that efficient and sustainable catalysis with titanocene complexes and epoxides as organic substrates is not restricted to epoxide hydrosilylations. The recently developed radical arylations of epoxides^[^ ^69^ ^]^ that constitute a highly appealing and substantially milder alternative to the classical Friedel‐Crafts alkylation is another example in this realm.

We are confident that based on the well documented activation of ketones^[^ ^70^ ^]^ and aziridines^[^ ^71^ ^]^ by titanocenes through electron transfer many other exciting sustainable and catalytic reactions for the preparation of important compounds will be developed in the near future.

## Conflict of Interest

The authors declare no conflict of interest.
